# The Importance of Catalytic Effects in Hot-Electron-Driven Chemical Reactions

**DOI:** 10.1021/acsnano.4c12923

**Published:** 2024-12-04

**Authors:** Farheen Khurshid, Jeyavelan Muthu, Yen-Yu Wang, Yao-Wei Wang, Mu-Chen Shih, Ding-Rui Chen, Yu-Jung Lu, Drake Austin, Nicholas Glavin, Jan Plšek, Martin Kalbáč, Ya-Ping Hsieh, Mario Hofmann

**Affiliations:** † Department of Low-Dimensional Systems, J. Heyrovský Institute of Physical Chemistry, Academy of Sciences of the Czech Republic, v.v.i., Dolejškova 3, 18223 Prague 8, Czech Republic; ‡ Department of Physics, National Taiwan University, Taipei 10617, Taiwan; § Research Center for Applied Sciences, Academia Sinica, Taipei 11529, Taiwan; ∥ Graduate Institute of Applied Physics, National Taiwan University, Taipei 10617, Taiwan; ⊥ Department of Electrical Engineering and Computer Sciences, Massachusetts Institute of Technology, Cambridge, Massachusetts 02139, United States; ◆ Institute of Atomic and Molecular Science, Academia Sinica, Taipei 10617, Taiwan; ○ Air Force Research Laboratory, Materials and Manufacturing Directorate, WPAFB, Ohio 45433, United States

**Keywords:** Gradient, Bimetallic Alloy, Compositional Catalyst Modification, Hot-Electron, Photocatalysis

## Abstract

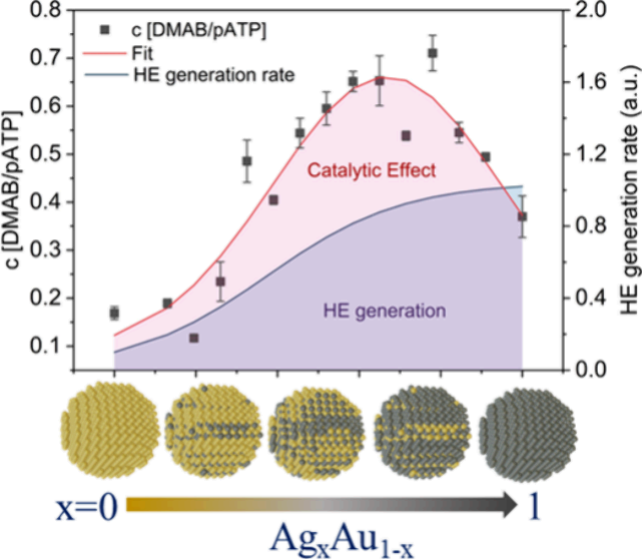

Hot electrons (HEs) represent out-of-equilibrium carriers that are capable of facilitating reactions which are inaccessible under conventional conditions. Despite the similarity of the HE process to catalysis, optimization strategies such as orbital alignment and adsorption kinetics have not received significant attention in enhancing the HE-driven reaction yield. Here, we investigate catalytic effects in HE-driven reactions using a compositional catalyst modification (CCM) approach. Through a top-down alloying process and systematic characterization, using electrochemical, photodegradation, and ultrafast spectroscopy, we are able to disentangle chemical effects from competing electronic phenomena. Correlation between reactant energetics and the HE reaction yield demonstrates the crucial role of orbital alignment in HE catalytic efficiency. Optimization of this parameter was found to enhance HE reaction efficiency 5-fold, paving the way for tailored design of HE-based catalysts for sustainable chemistry applications. Finally, our study unveils an emergent ordering effect in photocatalytic HE processes that imparts the catalyst with an unexpected polarization dependence.

## Introduction

Hot electrons (HEs) are out-of-equilibrium carriers with significant potential in chemistry.^1−3^ Due to their high energy compared to equilibrium statistics, they can carry out reactions that are unattainable under conventional conditions. Hot electrons are considered promising for conducting complex reactions with high selectivity and have been employed to degrade organic pollutants in wastewater,^4−7^ reduce CO_2_ through direct population of antibonding orbitals,^8−11^ and oxidize CO via oxygen activation.^12^ To enhance their impact in tailored and sustainable chemistry, significant effort is being invested to increase the efficiency of HE-driven reactions.^12^

Historically, the effectiveness and selectivity of catalytic processes have been ascribed to the energetics of reactant adsorption^13^ and the kinetics of reactant exchange on active sites.^14,15^ Surprisingly, these catalytic effects have not received significant attention in HE-driven reactions, and research efforts have instead focused on enhancing the overall HE emission yield through modification of the emitter^16^ especially through the utilization of electronic junctions.^17,18^

The limited advances in establishing the impact of catalytic processes in HE-driven reactions are due to challenges in disentangling catalytic effects from competing factors. Zheng et al. changed the HE catalyst’s surface and observed an increased photocatalytic HER reaction current but ascribed the enhancement to a coinciding variation of the emitter’s band structure.^18^ Dwivedi et al. identified a change in photocatalytic reactivity with metal doping,^19^ but changes in plasmonic HE generation efficiency also had to be considered.^20,21^ Finally, Lee et al. investigated an inverse problem by characterizing the generation of hot electrons during chemical reactions and observed HE current differences if an exposed oxide interface was introduced.^22^ Simultaneous changes in HE injection geometry, however, complicated the assignment of the source of enhancement.^17^

We here provide a clear demonstration of catalytic enhancement of HE-driven reactions and a detailed study of their origin through a compositional catalyst modification (CCM) technique. CCM is a powerful tool to modify the energetics of reactant adsorption and the distribution of reaction states.^23^ By employing a combination of high-throughput synthesis and comprehensive characterization methods, a wide range of catalytic behavior is observed. The monotonic dependence of the HE yield on composition agrees with previous theoretical predictions. Photodegradation experiments and electrochemical EQE measurements, however, both show an unexpected enhancement in catalytic activity at intermediate compositions. Through direct measurement of the redox levels in a model system, a clear correlation between the orbital alignment and the HE catalytic efficiency was identified. This effect was shown to dominate the HE-driven catalytic process over other HE effects, resulting in a 5-fold increase in catalytic efficiency at optimized conditions. The CCM geometry also provides exciting evidence of an emergent ordering effect in photocatalytic HE chemistry. Our approach demonstrates the potential of compositional optimization toward HE-driven chemistry with enhanced efficiency and unparalleled functionality.

## Result and Discussion

Compositional catalyst modification investigates the effect of electronic changes on the adsorption and catalysis upon introduction of a functional additive to a host material.^23^ To survey the large range of possible compositions, we utilized a high-throughput synthesis method. Different from conventional synthesis approaches that rely on the sequential fabrication of compositionally varying catalysts,^19,24^ our approach permits the simultaneous creation of catalysts with finely adjustable compositions at different locations on the same substrate. This method not only enhances the efficiency of the synthesis and characterization process but also minimizes sample-to-sample variations, as all processing is conducted at the same time.

**Figure 1:**
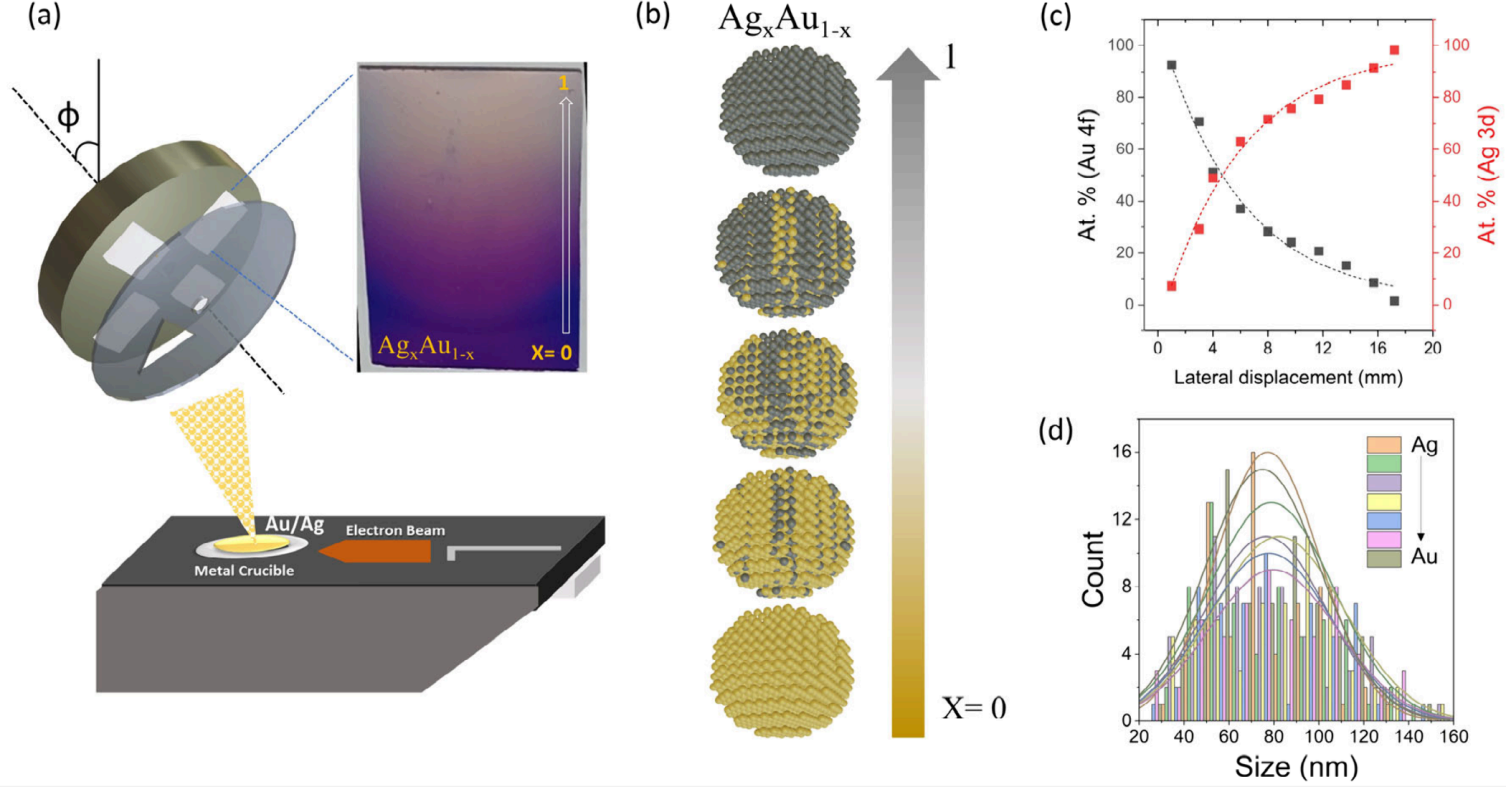
Concept of spatially controlled compositional catalyst modification (CCM). (a) Schematic of deposition process utilizing a rotating shadow mask that obstructs a portion of an electron-beam-induced metal atom flux hitting the sample; (inset) photograph of resulting sample that exhibits a spatially varying deposition thickness as evidenced by changing contrast on the SiO_2_ substrate. (b) Schematic representation of a gradient bimetallic alloy particle assembly at different locations on the sample that was obtained by sequentially depositing Ag and Au gradients. (c) Atomic concentration of both Au and Ag obtained by X-ray photoelectron spectroscopy and plotted as a function of lateral displacement. (d) Transmission-electron-derived histogram of alloy nanoparticle’s size distribution fitted with a Gaussian curve, which shows a similar center of weight independent of alloy composition.

The fabrication process relies on the physical vapor deposition process of two thin films that exhibit thickness gradients across the sample. By partial occlusion through a rotating shadow mask, gradually increasing film thicknesses of Au are deposited on Ag films with gradually decreasing thickness, thus resulting in the retention of the total film thickness but changing compositions between 0% and 100% relative Au concentration (Figure 1(a)). Subsequent annealing results in the formation of alloyed nanoparticles with finely controllable composition ratios at specific locations within a single sample, providing an ideal platform to investigate the influence of metal composition on hot electron generation (Figure 1(b)).

The controllable composition of these particles at different locations within the sample was confirmed by X-ray photoelectron spectroscopy (Figure 1(c)), and the surface sensitivity of the technique indicates that surface segregation is negligible.^25^

Microscopic characterization was conducted to evaluate the resulting morphology. Statistical high-resolution transmission electron microscopy reveals similar particle size distributions for all investigated alloy compositions (Figure 1(d)) and Supplementary Figure S2). Moreover, at the observed particle size the alloy exhibits bulk-like behavior,^26^ and all particles are expected to terminate into (111) surfaces.^27^

**Figure 2:**
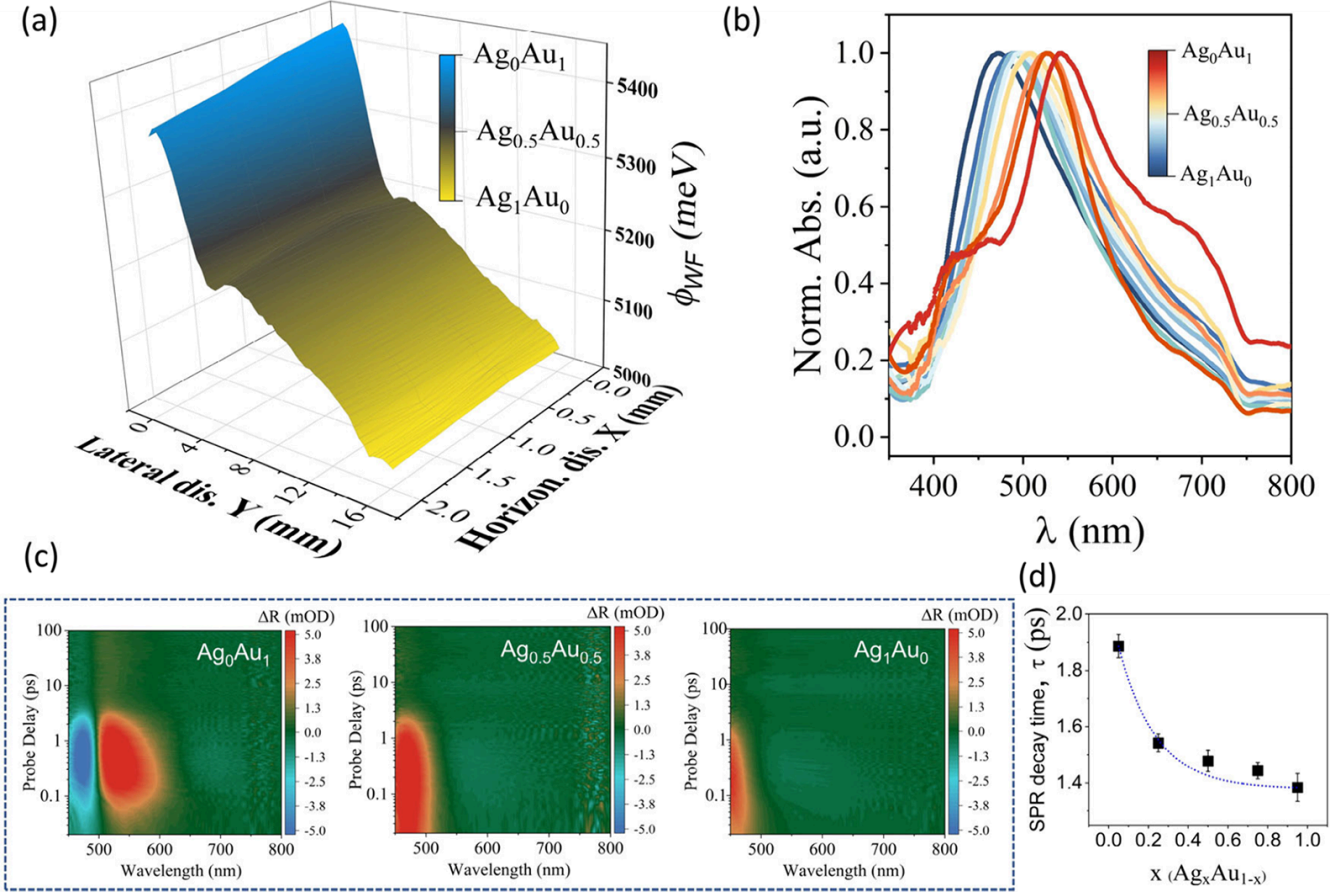
Electronic property characterization of alloy particles produced by CCM. (a) Spatially resolved Kelvin-probe measurements of the sample showing a decreasing work function in regions with increased Ag concentration; the color bar was derived from predicted compositions according to Figure 1(c)). (b) Local UV–vis absorption spectra at different sample locations corresponding to different compositions of Ag_*x*_Au_1–*x*_, showing a smoothly varying transition between pure Ag and Au plasmonic resonances. (c) Transient absorption spectra (TAS) with 400 nm pump and 450–800 nm probe of alloys with different Ag_*x*_Au_1–*x*_ compositions indicating an SPR decay feature around 550 nm. (d) Extracted SPR decay times vs composition (more details in Supporting Information).

The compositional variation imparts the catalyst with finely tuned electronic properties. Spatial Kelvin probe mapping (Figure 2(a)) shows a clear decrease in work function along the *y*-direction, which coincides with the direction of compositional variation. The axis perpendicular to the gradient direction, however, does not show such a dependence, which demonstrates the reliability of the CCM process. The observed direct correlation between work function and composition agrees with the predicted monotonic change of work functions between the extreme cases for pure Au catalyst and pure Ag dopant.^28,29^

Due to the use of two plasmonic materials, the monotonic variation of electronic properties can also be investigated by optical characterization techniques. We conduct localized UV–visible absorption spectroscopy at different sample locations that correspond to specific compositions (Figure 2(b)). A continuously varying surface plasmon resonance (SPR) is observed that shifts from 570 to 480 nm between pure Au and pure Ag. This observed variation agrees with the theoretical model proposed by Luca et al.,^30^ which only considers composition-induced changes in charge density. Moreover, the presence of a single SPR peak confirms the formation of a uniform alloy, as more complex morphologies, such as core–shell structures, would display two or more SPR peaks.^21^ Spatially selective absorption measurements furthermore demonstrate the smooth variation of absolute absorption strength across the sample, ruling out local maxima in scattering efficiency (Supplementary Figure S1).

The impact of compositional variation on the HE yield was investigated by femtosecond transient absorption spectral (TAS) analysis. We first utilized a 400 nm excitation pump (close to the plasmon resonance wavelength of Ag-rich alloys) and 450–800 nm probe wavelength. In the 2D-TAS profile (Figure 2(c), the negative signal of excited state absorption (ESA) at 475 nm was attributed to electron transitions from 5d to 6sp states.^31^ The positive signal at 550 nm signifies the occurrence of an interaction between photoexcited HEs and plasmonic oscillations.^32^ To quantify the occurrence of HEs, the temporal decay profile of this feature was plotted as a function of the composition ratio (Figure 2(d)). The decay time decreased exponentially with higher Ag ratios, indicating the faster relaxation of HEs through enhanced emission in Ag-rich phases.

A similar trend in relaxation time was also observed when exciting close to the plasmon resonance of Au-rich alloys (Figure S4(b) and Figure S4(c)), which highlights the utility of CCM for optimizing HE emission efficiency independent of plasmonic absorption efficiency. This behavior furthermore confirms the direct dependence of HE electron yield on Ag concentration, due to its more suitable band structure compared to Au.

The agreement of diverse experimental results demonstrates the potential of CCM to smoothly adjust the electronic properties of the HE emitter between the two pure conditions. The observed monotonic dependence of work function, plasmon resonance, and HE yield on Ag relative concentration corroborate that all these parameters can be captured by a noninteracting dispersion of two atomic components.

**Figure 3:**
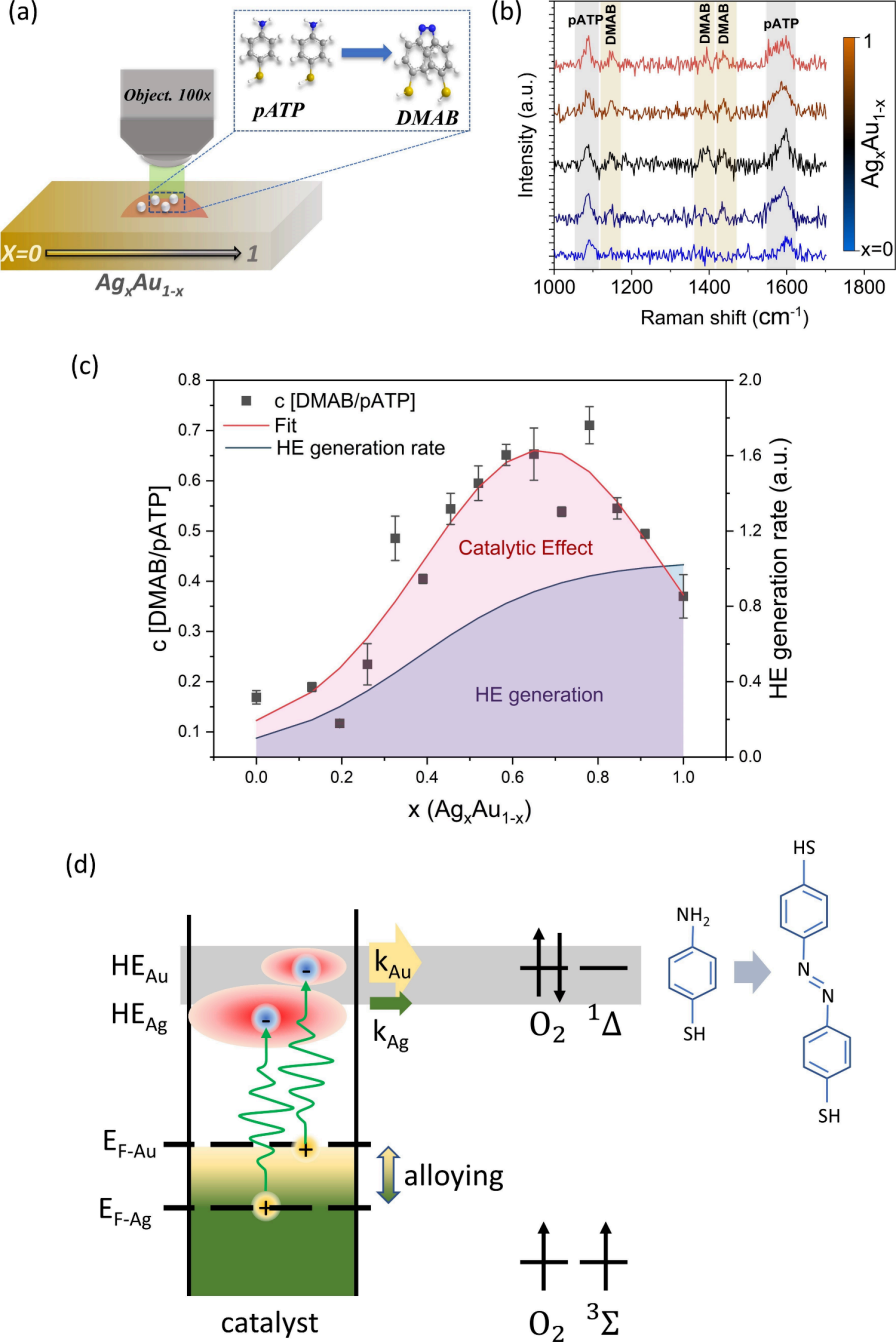
Photocatalytic degradation under variable catalyst composition. (a) Depiction of photocatalytic conversion experiment between pATP and DMAB t. (b) Representative SERS spectra at different compositions with an indication of Raman peaks corresponding to the pATB precursor and DMAB reaction product. (c) Measured photocatalytic conversion efficiency as a function of composition (left *y*-axis) with predicted HE generation rate (right *y*-axis) from plasmonic decay times obtained in Figure 2(d) (derivation in the Supporting Information). (d) Schematic of HE-catalyzed oxygen activation from the triplet to the singlet state with an indication of different Fermi level (*E*_F_), hot electron yield (HE), and heterogeneous transfer rate (*k*) for Au- and Ag-rich catalysts.

Surprisingly, this noninteraction picture is not sufficient to explain the catalytic efficiency under CCM. The hot-electron-mediated conversion of para-amino thiophenol (pATP) to *p*,*p*′-dimercaptoazobenzene (DMAB) was chosen as a model system (Figure 3(a)) due to its attractive features for our CCM studies. First, the reaction exhibits qualitatively similar kinetics on Au and Ag surfaces due to similar physisorption energies,^33^ adsorption geometries, and reaction pathways.^34^ Moreover, the reactants have distinct Raman signatures (Figure 3(b)) that permit in situ characterization of the reaction process under illumination.^35^

Extensive previous work on the reaction process has put forward an optically activated oxidation process as the underlying mechanism. Oxygen is adsorbed on the catalyst and is activated through a hot-electron-mediated activation of the oxygen singlet, which induces the conversion from pATP to DMAB.^36^ Consequently, the conversion process acts as a sensitive probe for the presence of activated oxygen radicals. DFT calculation demonstrated that the adsorption energies of oxygen on both surfaces are comparable,^37,38^ suggesting that changes in conversion efficiency are not due to differences in the adsorption kinetics. Moreover, the pATP photodimerization was shown to be relatively insensitive to photothermal effects,^36^ which we confirmed by power-dependent SERS measurements (Figure S8).

Under 532 nm excitation, the photocatalytic conversion efficiency is calculated as the ratio of produced DMAB to remaining pATP (Figure 3(c)). When the variation in HE catalytic conversion efficiency between pure Au and Ag composition is compared, a 3-fold increase in conversion efficiency is observed (Figure 3(c)). This enhancement agrees with the increased HE yield for Ag described in the previous section.

Unexpectedly, a notably enhanced catalytic efficiency is identified for intermediate Ag concentrations with Ag_0.65_Au_0.35_ exceeding the conversion efficiency of pure Au 5-fold (Figure 3(b)). To confirm that this enhancement is not only due to plasmonic absorption effects, we duplicate the photodegradation measurements with a 488 nm excitation source, which is resonant with Ag plasmons. Furthermore, we normalize the catalytic activity relative to the Ag metal surface concentration, as obtained by XPS (Figure S5(b)). Both approaches confirm that the catalytic efficiency is highest at the intermediate composition of Ag_0.65_Au_0.35_ (Figure S6), indicating that the CCM enhanced activity is robust and independent of optical properties.

The observed reactivity enhancement at intermediate compositions cannot be explained by changes in the HE yield since they exhibit a monotonic dependence, as described earlier. To illustrate this discrepancy, we contrast the reactivity with theoretical predictions for the hot electron generation rate (see Supporting Information for more details). We can identify a significant deviation between the two processes (Figure 3(c)).

Instead, the non-monotonic catalytic enhancement emphasizes the importance of the overlap between carrier states and orbitals in HE catalysts. As the oxygen adsorbate energy levels coincide with the energy distribution of photoexcited electrons, a significantly increased electron transfer rate from the catalyst into the adsorbate orbital is expected. Consequently, the adjustable HE energy distribution in Ag/Au alloys provides a route to controlling the overlap and improving the reaction rate (Figure 3(d)). To further illustrate this capability, we also conduct photocatalytic CO_2_ reduction reactions and observe a pronounced dependence of the CO yield on composition (Supplementary Figure S10).

**Figure 4:**
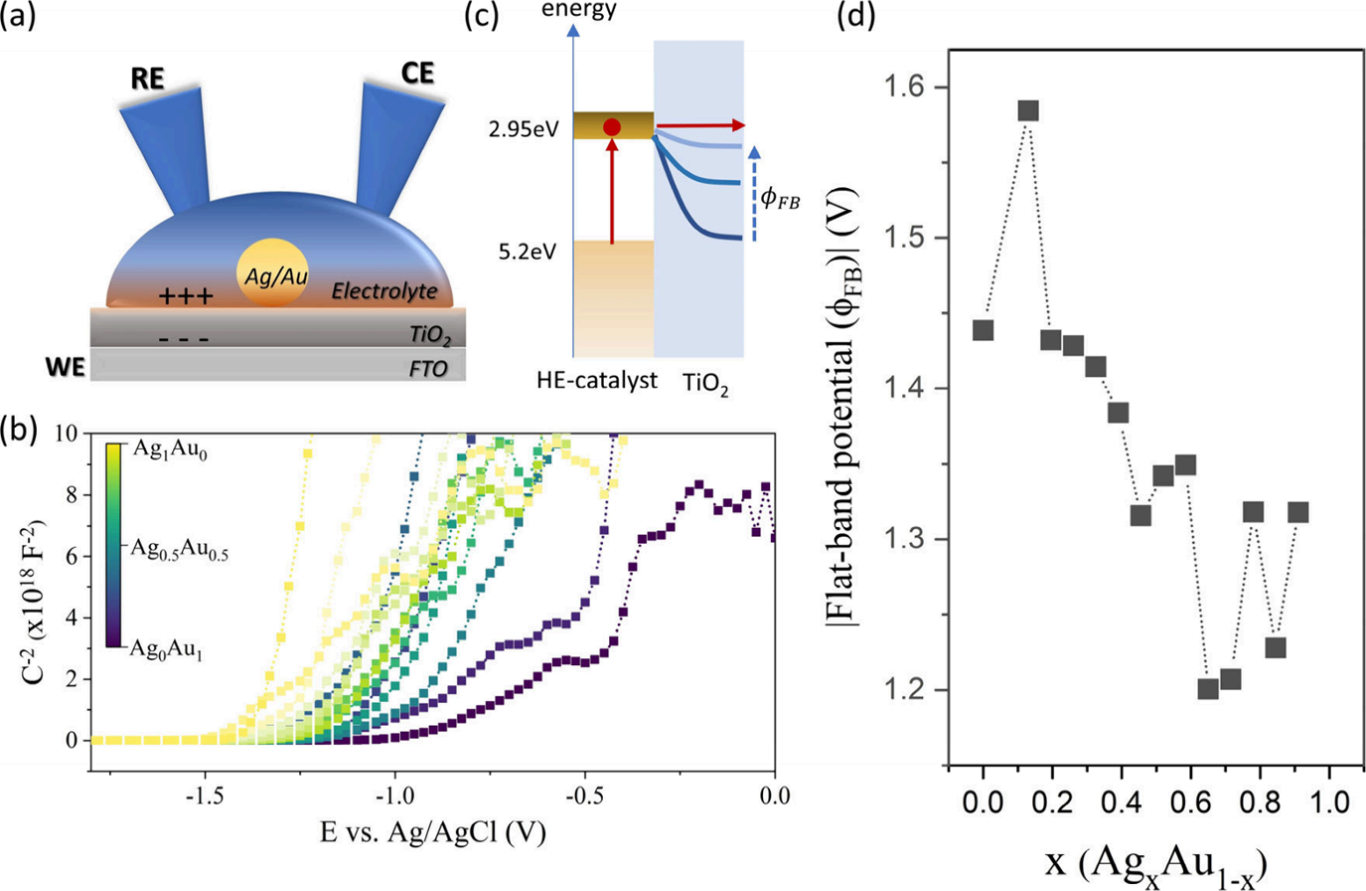
Electrochemical determination of emitter band alignment. (a) Schematic diagram of a heterojunction system where the Ag/Au HE emitter is brought in contact with an electrolyte-gated TiO_2_ film solid-state injection system with electrochemical gate. (b) Mott–Schottky plot of inverse junction capacitance vs applied electrochemical potential for varying HE emitter compositions. (c) Schematic representation of interfacial band alignment at different flat-band potential values (ϕ_*FB*_). (d) Absolute flat-band potential as a function of alloy composition.

We further confirm this non-monotonic dependence of HE reaction efficiency on composition by conducting systematic heterojunction measurements under local electrochemical gating. For this purpose, the CCM nanoparticles were brought into contact with a TiO_2_ film to form a heterojunction. Then, an electrolyte was employed to provide charges to the interface and locally gate the heterojunction structure (Figure 4(a)). We utilized an AC measurement technique to determine the capacitance of the heterojunction and visualize it in a Mott–Schottky plot (Figure 4(b)). A quasi-linear region is observed that decreases with smaller potentials, indicating a decrease in charge accumulation at the TiO_2_ interface that eventually terminates at the flat band potential.^39^ The flat band potential provides us with an estimate of the energy difference between the Fermi level of TiO_2_ and the HE emitter.^40^ When plotting the flat band potential as a function of emitter composition, we observe that the value remains negative, indicating that the transition from Ag- to Au-rich alloys does not change the sign of the band bending in TiO_2_, as previously observed^41^ and suggesting the presence of surface states on the TiO_2_.

More importantly, we see that the absolute of the flat band potential exhibits a non-monotonic variation with composition and a minimum around *x* = 0.65 (Ag_0.65_Au_0.35_) (Figure 4(d)). This observation corroborates our hypothesis that the emitter composition modifies the barrier between HE emitter and reactant (in this case TiO_2_).^42^

The utilization of an electrolyte with a well-defined reference electrode^43^ permits the extraction of an absolute value of the injection barrier edge. Converting the flat band potential from Ag/AgCl to vacuum conditions suggests a TiO_2_ conduction band position of 2.95 eV. Given the wide range of surveyed HE emitter work functions, this level represents the optimal overlap between hot electron distribution and reactant levels (Figure 4(c)). At optimal composition, Figure 2(a) reveals an HE emitter work function of 5.2 eV, which indicates an energy difference between the HE emitter Fermi level and hot electron states of 2.3 eV. This energy difference agrees well with the expected energy range for photoexcited electrons under a 532 nm excitation source (2.33 eV).

**Figure 5:**
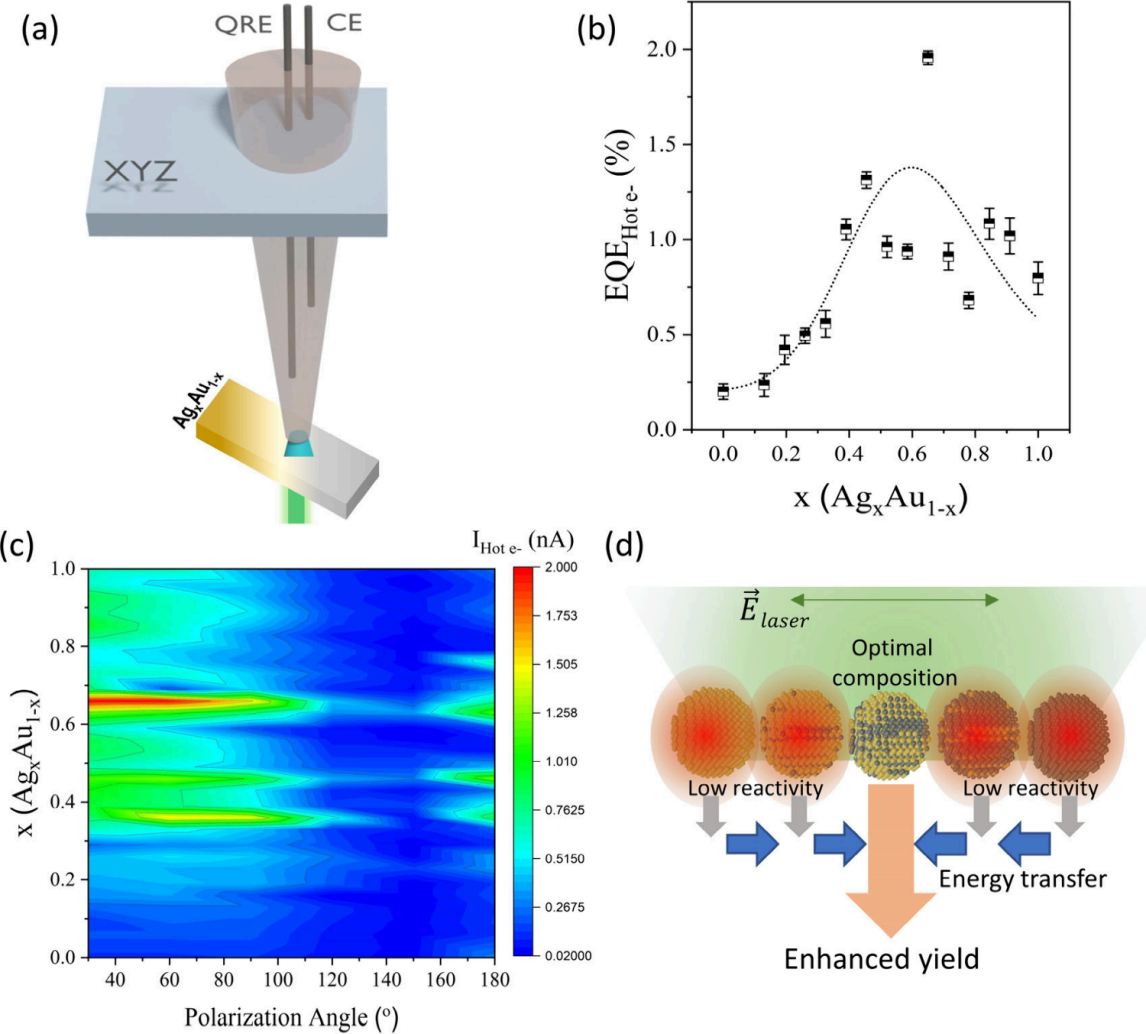
Photoelectrochemical HE characterization. (a) Schematic representation of the localized photoelectrochemical analysis system consisting of an electrolyte-filled (0.1 M Na_2_SO_4_) capillary with quasi-reference and counter electrode that touches the FTO/TiO_2_/Ag_*x*_Au_(1–*x*)_ sample, which is back-illuminated with a 532 nm laser. (b) Extracted external quantum efficiency (EQE) of hot electron injection into the electrolyte vs alloy composition. (c) Polarization-dependent photocurrent mapping that demonstrates a pronounced polarization effect at optimized compositions. (d) Schematic representation of the mechanism responsible for the observed polarization response: Plasmonic energy transfer from less reactive particle compositions to highly reactive compositions leads to enhanced yield if the light *E*-field is parallel to the gradient direction.

Finally, we combine photocatalytic and electrochemical measurement systems to quantify the impact of HE catalysis on the overall reaction yield. For this purpose, a bottom laser illumination (λ = 532 nm) is integrated with a microdroplet-based electrochemical setup (Figure 5(a)). This arrangement provides photoelectrochemical characterization of hot electrons with high spatial resolution, which is determined to be ∼30 μm and depends on both the size of the microdroplet and the illumination area.

We calculate the external quantum efficiency (EQE) of hot electrons from the difference in electrochemical current under illumination compared to the dark background current (details are provided in the Supporting Information). This electrochemical photocurrent is plotted as a function of the emitter composition in Figure 5(b). We observe an increase in EQE with a maximum at *x* = 0.65 (Ag_0.65_Au_0.35_), in agreement with both photocatalytic and heterojunction measurements. Moreover, we demonstrate that thermophotonic effects have limited impact on our EQE analysis by power-dependent measurements (Figure S7).

The correlation of three different measurement techniques demonstrates the importance of the alignment between HE emitter levels and the reactant’s redox states in controlling the HE reaction efficiency.

The presented sample geometry not only provides a powerful tool for applying CCM toward optimizing the yield of HE chemistry but also creates an emergent phenomenon. Due to the gradient alloying process, the sample exhibits an anisotropic composition distribution with one direction having uniform composition and one direction showing variable composition.

To evaluate the impact of this compositional anisotropy on the HE reactivity, we conduct polarization-dependent EQE measurements. A pronounced effect of the light polarization on EQE is observed with a 6-fold enhancement in EQE when the *E*-field is parallel to the axis of compositional change (Figure 5(c)).

This effect can be understood by the competition of plasmonic coupling and EQE in particle assemblies. For particles with low HE EQE, the plasmonic loss is decreased and energy transfer between neighboring particles occurs parallel to the *E*-field.^44^ However, if the EQE is high, plasmonic loss through HE emission dominates, and no energy transfer is expected. In our assemblies of particles with spatially varying compositions, highly reactive regions would exhibit an enhanced HE yield by coupling with lower reactivity regions (Figure 5(d)).

Due to the anisotropy of the sample, such a coupling will occur only in the direction of the composition gradient, resulting in an enhanced polarization as observed in our experimental results.

## Conclusions

In summary, we demonstrate the importance of orbital alignment between emitters and reactants in enhancing the effectiveness of HE-driven chemical reactions. By utilizing a powerful compositional variation approach, high-throughput characterization of catalysts with widely varying electronic properties is achieved. Electrochemical and heterojunction measurements unambiguously demonstrate the controlling effect of catalytic effects in the HE reaction process, whereas plasmonic contributions are found to be secondary. Upon optimization, the HE-driven reaction performance could be enhanced 5-fold, as evidenced by photodegradation and photoelectrochemical techniques. Compositionally graded HE catalysts furthermore exhibit a polarization dependence that arises from the synergistic effects of plasmonic focusing and reaction selectivity and could be employed in future reaction schemes that exploit the quantum nature of light.

## Methods

### Bimetallic Gradient Alloy Fabrication

The bimetallic gradient alloy sample was prepared on a substrate by using an electron beam evaporation system (ast Peva-600I) with a stage that is rotating at a 45° tilt angle. To achieve the gradient, we utilized a specially designed shadow mask placed on the rotating stage with a spacing of 12 mm. The evaporation was carried out at a rate of 0.1 A s^–1^ under a high vacuum (<2 × 10^–6^ Torr). The dewetting of the thin film sample was performed at an annealing temperature of 350 °C.

### Optical and Morphological Characterizations

Plasmonic absorption spectra were measured using a home-built micro-spectrophotometer. A collimated white light (400–1000 nm) from a halogen lamp (Ocean Optics, HL-2000-HP) impinges on the nanostructure through an objective lens (20×, NA 0.25) at normal incidence. The transmitted spectra were recorded using a fiber-coupled spectrum analyzer (B&W Tek, BRC711E) and normalized to the transmission of a bare FTO-coated glass substrate. Transmission electron microscopic (TEM) analyses were conducted using a JEOL JEM-2100F with an acceleration voltage of 200 kV. The TAS was performed by using a FemtoFrame II transient absorption spectrometer, a commercially available system by IB Photonics. The instrument offers a temporal instrument response function of 150 fs. A Ti:sapphire femtosecond laser source (OPA (TOPAS-C)) was used to produce pulses with a duration of 100 fs and operates at a repetition rate of 1 kHz. The pump and probe beams were characterized by spot sizes of approximately 7 × 10^–4^ cm^2^ and 1.6 × 10^–4^ cm^2^, respectively. To generate the pump beam (wavelengths: 400 and 532 nm, duration: 100 fs, repetition rate: 1 kHz), we employed a Ti:sapphire laser combined with an OPA system. On the other hand, a broadband white-light probe beam spanning from 450 to 800 nm and featuring a pulse width of 30 fs was generated using supercontinuum generation. Work function mapping was performed using a Kelvin probe microscope (KP Technology). The measurements were performed in real time using a Digital TFT oscilloscope, which offered a resolution of 1–3 meV. The morphology of the Ag–Au gradient alloy sample was examined using scanning electron microscopy (JSM 6500F). XPS measurements were carried out using a VG ESCA3MkII electron spectrometer under a base pressure higher than 10^–9^ mbar. Al Kα radiation was used for the excitation of the electrons. The binding energies were referenced to the binding energies of Ag 3d and Au 4f electrons.

### Local Scanning Photocurrent and Electrochemical Measurements

A micro-electrochemical cell was employed (see Supporting Information) as a tool for conducting local scanning photocurrent measurements on electrodes composed of bimetallic gradient alloys. The configuration of the cell included a quasi-reference electrode based on Ag/AgCl and a Pt counter electrode. To ensure the integrity of the sample, the micro-electrochemical cell was positioned at a distance of roughly 10 μm above the Ag/Au electrode. This positioning was accomplished with the assistance of an optical microscope and a micropositioner, minimizing the risk of sample damage. Prior to scanning the localized photocurrent measurements, an amperometric *i*–*t* measurement was performed in order to establish the liquid contact on the sample surface. The electrolyte flow rate was set to 50 μL/min using a syringe pump. The photocurrent was recorded as a function of different polarization angles in 0.1 M Na_2_SO_4_ with a scan rate of 10 mV/s. Electrochemical impedance spectra (EIS) were recorded over a frequency range spanning from 1 Hz to 1 MHz, utilizing a perturbation voltage amplitude of 50 mV. A Nyquist curve was fitted by using an equivalent circuit, enabling the estimation of solution resistance (*R*_s_) and capacitance (*C*_dl_). This information was critical to understanding the interfacial charge transfer properties of the Ag_*x*_Au_1–*x*_ system.

### In Situ Photocatalysis

A 10 mM solution of pATP was prepared by dissolving it in 50 mL of ethanol (as the solvent). Then the sample was immersed in the pATP solution and allowed to adsorb pATP for 5 h under dark conditions. Afterward, the alloy sample was thoroughly washed with ethanol to remove any unbound pATP. Finally, the alloy sample was exposed to visible light for 5 min before Raman spectroscopy measurements. The Raman spectra were collected at room temperature under ambient air using excitation wavelengths of 488 nm (Jobin Yvon Horiba Xplora) and 532 nm (Jobin Yvon Horiba), with a laser power of 150 μW. The low laser power and short exposure times were chosen to ensure that the conversion was not limited by the adsorption process (more details in the Methods section).

## Supporting Information Available

In-depth characterization of gradient samples, including UV–visible absorption, transmission electron microscopy, raw XPS spectra, transient absorption spectroscopy, Kelvin-probe microscopy; detailed photocatalytic SERS analysis and evaluation of thermophonic effects; theoretical calculation of hot electron yield, electrochemical impedance spectra, and comparison of photocatalytic CO_2_ reduction yields (PDF)
