# Supplementary material for: The Importance of Catalytic Effects in Hot-Electron-Driven Chemical Reactions
Source: ACS Nano. 2024 Dec 4;18(50):34332–40. doi: 10.1021/acsnano.4c12923 (PMC11656843; doi:10.1021/acsnano.4c12923)
Supplement: Supplementary file 1 — nn4c12923_si_001.pdf [file nn4c12923_si_001.pdf]

Supporting Information

The Importance of Catalytic Effects in Hot-Electron-Driven Chemical Reactions

*Farheen Khurshid,[1,2] Jeyavelan Muthu,[1,2] Yen-Yu Wang,[3] Yao-Wei Wang,[1] Mu-Chen Shih,[4] Ding-Rui Chen,[5,6] Yu-Jung Lu,[2,3] Drake Austin,[7] Nicholas Glavin,[7] Jan Plšek,[2] Martin Kalbáč,[2] Ya-Ping Hsieh,[6] and Mario Hofmann,[1]**

[1]Department of Physics, National Taiwan University, Taipei 10617, Taiwan

[2]Department of Low-Dimensional Systems, J. Heyrovský Institute of Physical Chemistry, Academy of Sciences of the Czech Republic, v.v.i., Dolejškova 3, 18223 Prague 8, Czech Republic

[3]Research Center for Applied Sciences, Academia Sinica, Taipei 11529, Taiwan

[4]Graduate Institute of Applied Physics, National Taiwan University, Taipei 10617, Taiwan

[5]Department of Electrical Engineering and Computer Sciences, Massachusetts Institute of Technology: Cambridge, US

[6]Institute of Atomic and Molecular Science, Academia Sinica, Taipei 10617, Taiwan

[7]Air Force Research Laboratory, Materials and Manufacturing Directorate, WPAFB, OH 45433, USA

*Correspondence to: Mario@phys.ntu.edu.tw

1. Optical absorption mapping of gradient bimetallic alloy (Ag_xAu_{1-x})

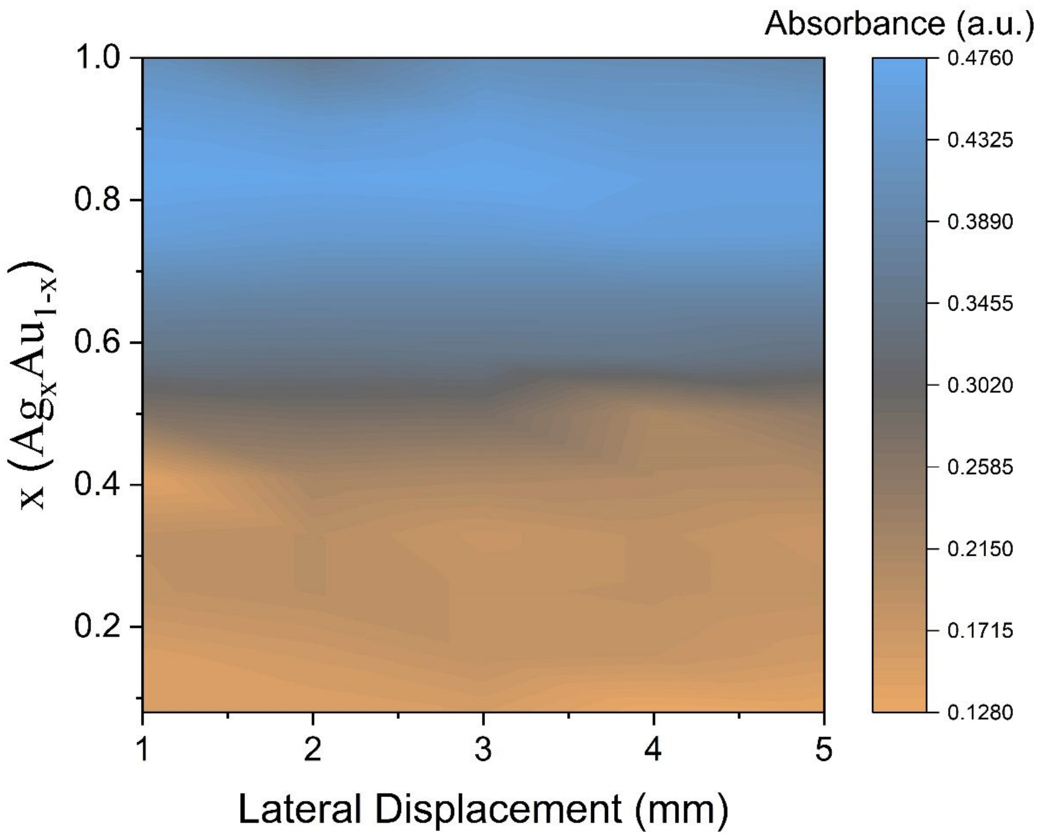

Figure S1. Local absorbance mapping of bimetallic gradient alloy nanoparticles on FTO substrate.

2. HR-TEM of gradient bimetallic alloy (Ag_xAu_{1-x})

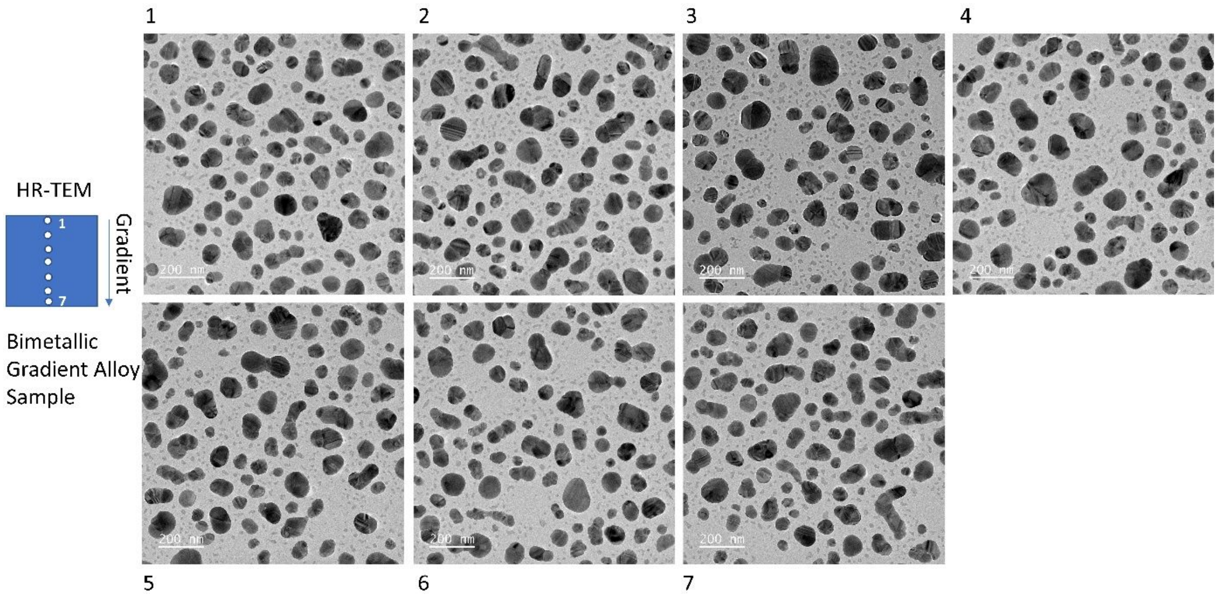

Figure S2. HR-TEM image of bimetallic gradient alloy nanoparticles.

High-resolution transmission electron microscopy (HR-TEM) analyses were carried out to examine the particle size and crystal orientation across different alloy compositions. The HR-TEM images (Figure S2 and the corresponding histograms (Figure 1(d)) reveal that the alloy particles exhibit a uniform size distribution across the various alloy compositions. These findings indicate that the particles are indeed similar in size, regardless of the specific alloy ratio. This uniformity in particle size and crystal structure ensures that the observed catalytic performance and hot carrier dynamics are not influenced by variations in these parameters.

3. X-ray photoelectron spectroscopy (XPS) of gradient bimetallic alloy (Ag_xAu_{1-x})

(a)

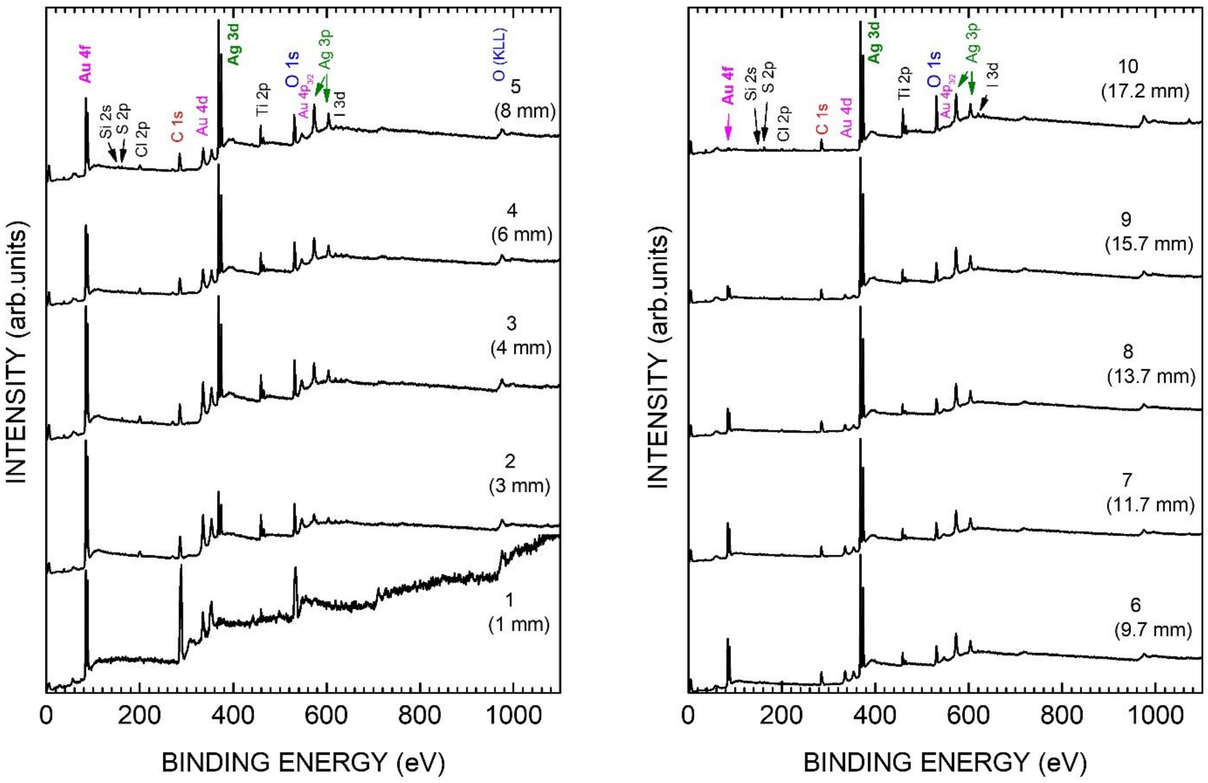

(b)

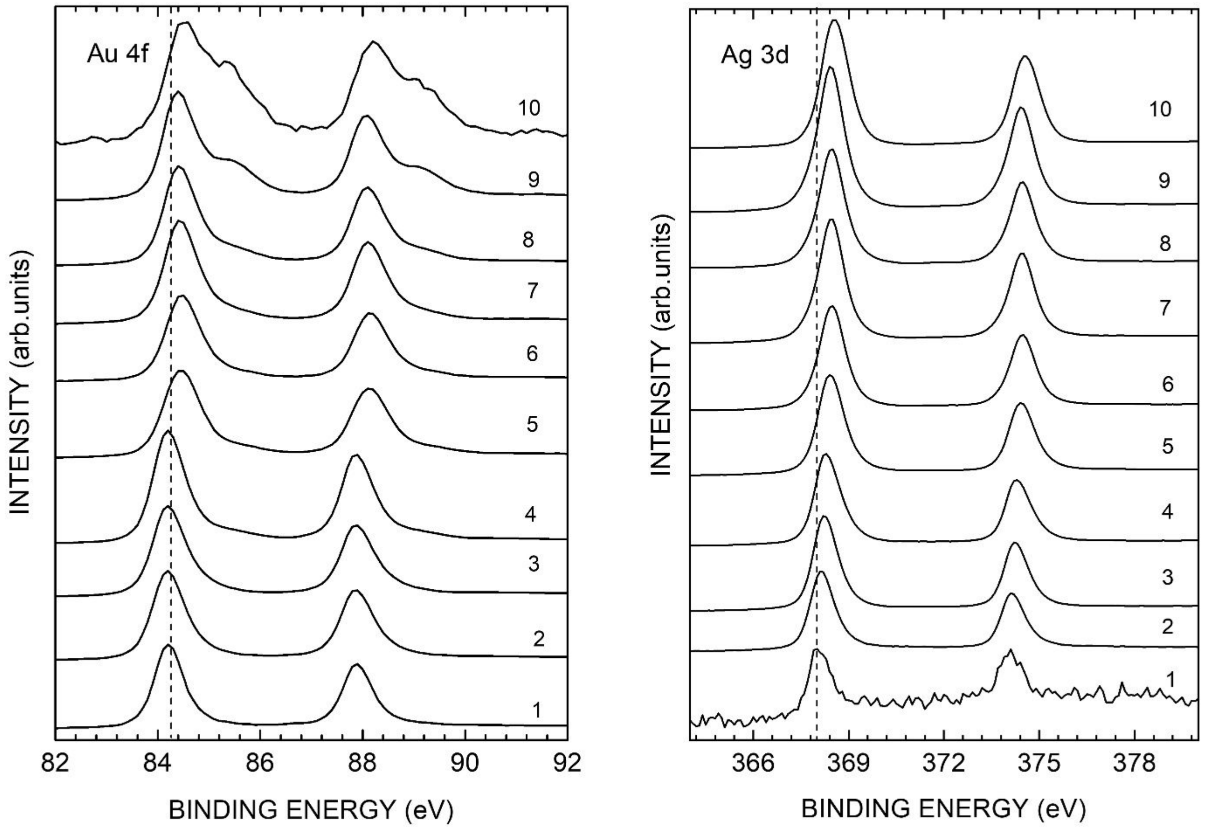

(c)

Sample	mm	No	x	I(Au4f)	I(Au4f)/Scof.	B.E. (Au4f)	I(Ag3d)	I(Ag3d)/Scof.	B.E. (Ag3d)	Int.suma(Au+Ag)	at. % (Au4f)	at. % (Ag3d)
AuAg	**1**	1	-4.5	8755	**485**	84.28	649	38	368.10	523.25	**92.75**	**7.25**
	**3**	2	-2.5	146669	**8130**	84.21	57447	3356	368.19	11485.80	**70.79**	**29.21**
	**4**	3	-0.5	133695	**7411**	84.21	121835	7117	368.27	14527.57	**51.01**	**48.99**
	**6**	4	1.5	97525	**5406**	84.24	157617	9207	368.28	14612.64	**37.00**	**63.00**
	**8**	5	3.5	86672	**4804**	84.47	208522	12180	368.44	16984.41	**28.29**	**71.71**
	**9.7**	6	-4	76672	**4250**	84.55	228081	13322	368.53	17572.58	**24.19**	**75.81**
	**11.7**	7	-2	73272	**4062**	84.54	265735	15522	368.49	19583.56	**20.74**	**79.26**
	**13.7**	8	0	44882	**2488**	84.44	239622	13997	368.49	16484.51	**15.09**	**84.91**
	**15.7**	9	2	26933	**1493**	84.44	275624	16100	368.49	17592.45	**8.49**	**91.51**
	**17.2**	10	3.5	3572	**198**	84.54	211469	12352	368.57	12550.13	**1.58**	**98.42**

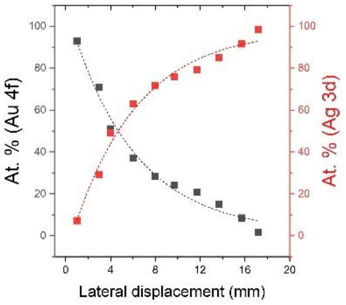

Figure S3. X-ray photoelectron spectra (XPS), (a) Survey Spectra of bimetallic gradient alloy nanoparticles. (b) Au and Ag high-resolution spectra of bimetallic gradient alloy nanoparticles. (c) Atomic concentration of both Au and Ag, and plotted as function of lateral displacement.

XPS measurements were carried out using a VG ESCA3 MkII electron spectrometer under a base pressure higher than 10^{-9} mbar. AlK_{α} radiation was used for the excitation of electrons. The binding energies were referenced to the binding energy of Ag 3d and Au 4f electrons. Figure S3(a) displays XPS spectra of bimetallic gradient on TiO_2 coated FTO substrate. The atomic concentration of both Au and Ag estimated from the high-resolution spectra (Figure S3(b)). Figure S3(c) displays the continuous variation of atomic concentration of Au and Ag confirming the formation of alloyed nanoparticles with finely controllable composition ratios within a single sample.

4. Transient absorption spectra (TAS) of gradient bimetallic alloy (Ag_xAu_{1-x})

(a)

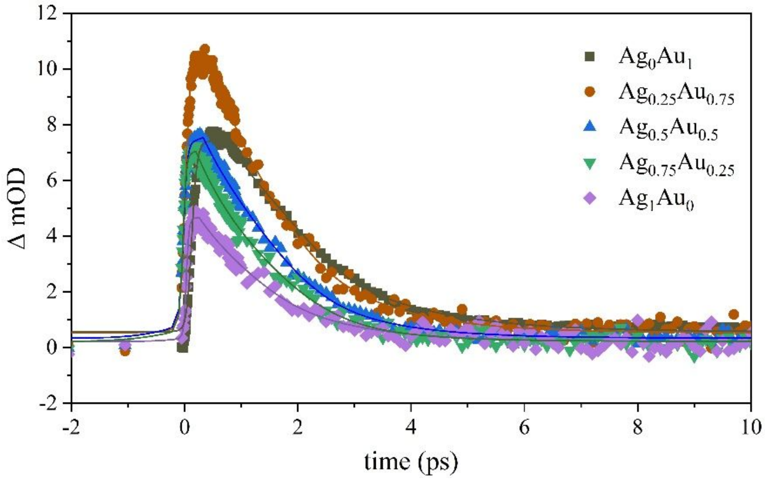

(b)

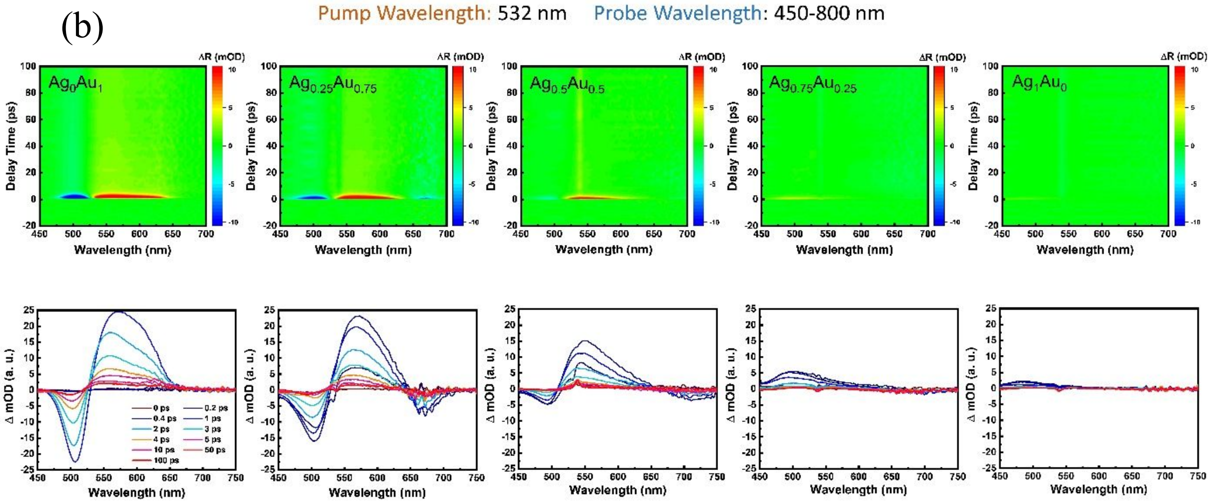

(c)

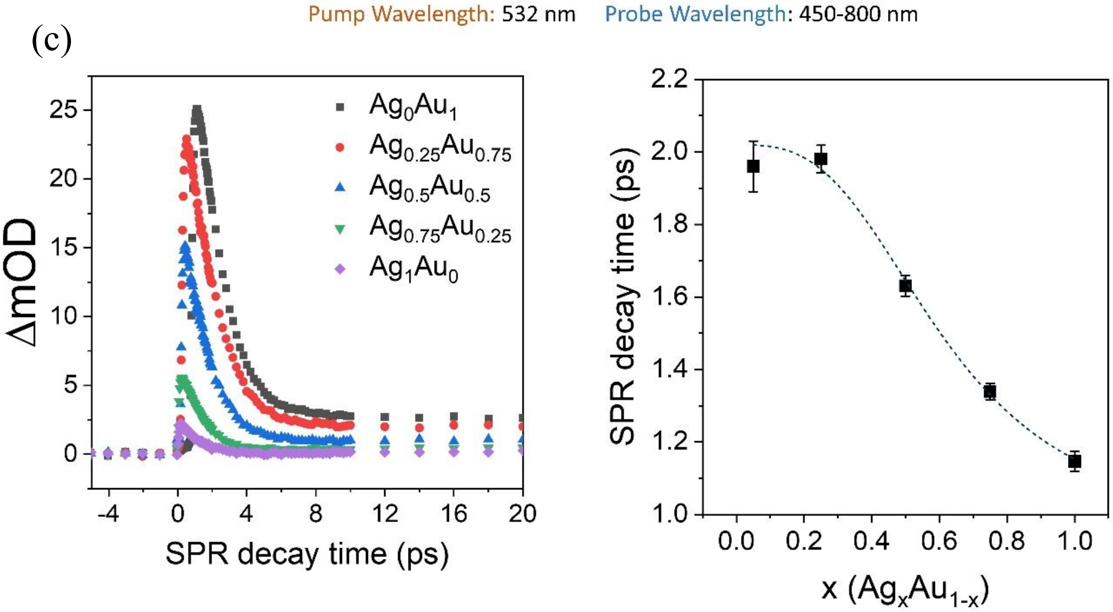

Figure S4. Transient absorption spectroscopy (a) SPR decay time (measured under 400 nm pump excitation) as a function of alloy compositions. (b) TAS spctra of bimetallic alloys measured under 532 nm pump excitation. (c) SPR decay time as a function of alloy compositions.

Transient absorption spectroscopy (TAS) measurements under conditions were performed more closely aligned with the surface plasmon resonance (SPR) maxima of the various alloy compositions. Specifically, we utilized a 532 and 488 nm pump, and a 450-800 nm probe wavelength range for these measurements. For, 400 nm pump wavelength, the decay time decreased exponentially with higher Ag ratios (Figure S4(a)), indicating the faster relaxation of HEs through enhanced emission in Ag-rich phases.

From the 532 nm pump TAS spectra (Figure S4(b)), we evaluated the SPR decay times across different alloy compositions. Consistent with our previous observations using 400 nm pump excitation, the decay time decreases as the Ag ratio increases (Figure S4(c)). This consistent trend across different excitation wavelengths corroborates our initial findings and further substantiates the observation that higher Ag content leads to faster relaxation of hot electrons (HEs) through enhanced emission in Ag-rich phases.

5. ***In-situ*** **photocatalytic Raman spectra of gradient bimetallic alloy (Ag_xAu_{1-x})**

(a)

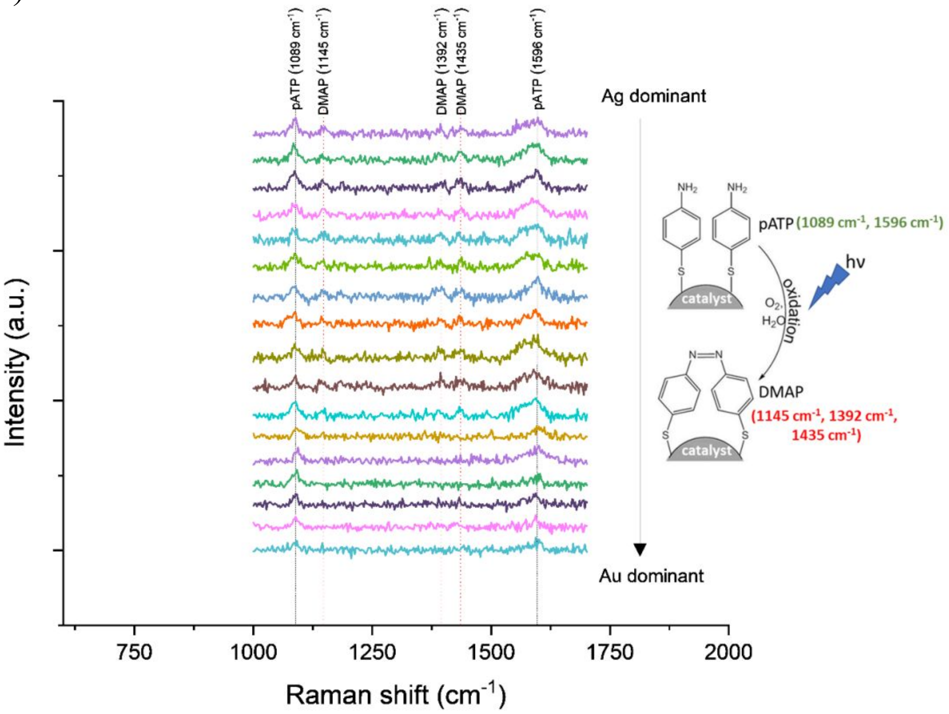

(b)

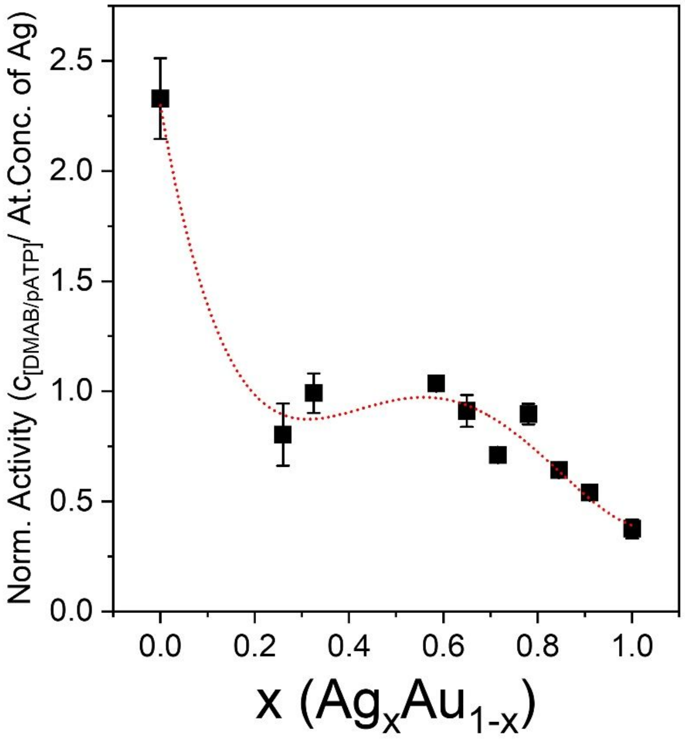

Figure S5. (a) Raman Spectra that corresponds to the plasmonic-hot electron-assisted photocatalytic conversion of pATP into DMAB under 532 nm excitation wavelength. (b) Normalized photocatalytic activity as function of different alloy compositions.

We investigate the hot-electron mediated photocatalytic conversion of para-amino thiophenol (pATP) to p,p'-dimercaptoazobenzene (DMAB).[1] In situ photocatalytic Raman experiments were recorded during conversion (Figure S5(a)) under 532 nm excitation. The recorded spectra exhibit characteristic pATP peaks at ~1089, and 1145 cm^{-1}, associated with the benzene ring bending mode, and C–S (nC–S) and C-N (nC–N) stretching modes, respectively. Additionally, peaks at 1392, 1345, and 1596 cm^{-1} correspond to the C–N and N=N stretching vibrational modes of DMAB.[2,3]

We normalized the catalytic activity relative to the Ag metal surface concentration (Figure S5(b)). The results of this normalization process indicate that the overall catalytic trend remains largely unchanged. This suggests that while the metal surface concentration does indeed influence catalytic activity, the enhanced catalytic performance observed at the specific alloy composition of $Ag_{0.65}Au_{0.35}$ is not solely attributable to composition dependent hot-elelctron generation.

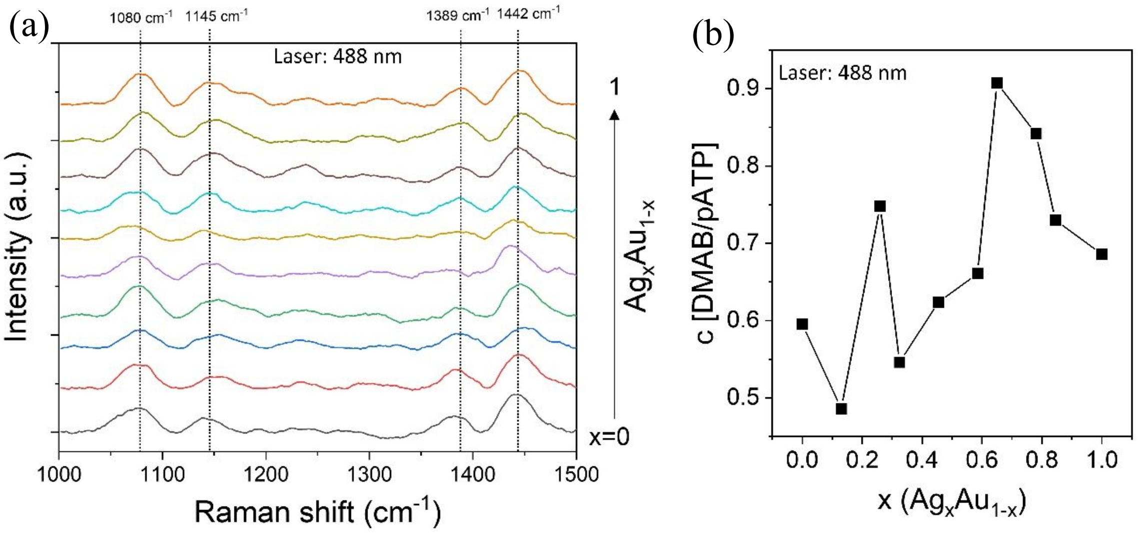

Figure S6. (a) Raman Spectra that corresponds to the plasmonic-hot electron-assisted photocatalytic conversion of pATP into DMAB under 488 nm excitation wavelength. (b) Photocatalytic conversion as function of different alloy compositions.

We have conducted additional photocatalytic experiments to ensure a more equitable comparison across different alloy compositions. We performed photocatalytic measurements using a 488 nm excitation wavelength, which is closer to the excitation wavelength of Ag-rich alloys (Figure S6(a)). This is in addition to our previous measurements conducted at 532 nm, which are more suitable for Au-rich alloys. Our results indicate that, similar to the findings with 532 nm excitation, the catalytic efficiency is highest at the intermediate composition of $Ag_{0.65}Au_{0.35}$ when using 488 nm excitation (Figure S6(b)). This suggests that the enhanced catalytic activity at this specific alloy ratio is robust and consistent across different excitation wavelengths.

6. Evaluation of thermophotonic effects

Previous work has demonstrated that heating could contribute to the yield of plasmonic reactions.[4] To rule out this effect, we conduct power dependent photocurrent measurements (Figure S7(a)) on the Au rich part. Thermal effects are expected to produce a saturation behavior. Instead, the photocurrent follows a power-law dependence (Figure S7(b)) throughout the measured range, that has been attributed to plasmonic emission of hot electrons.[5] This trend suggests that the low power employed and the good thermal contact of the nanoparticles with the substrate limit the effect of heating.[6]

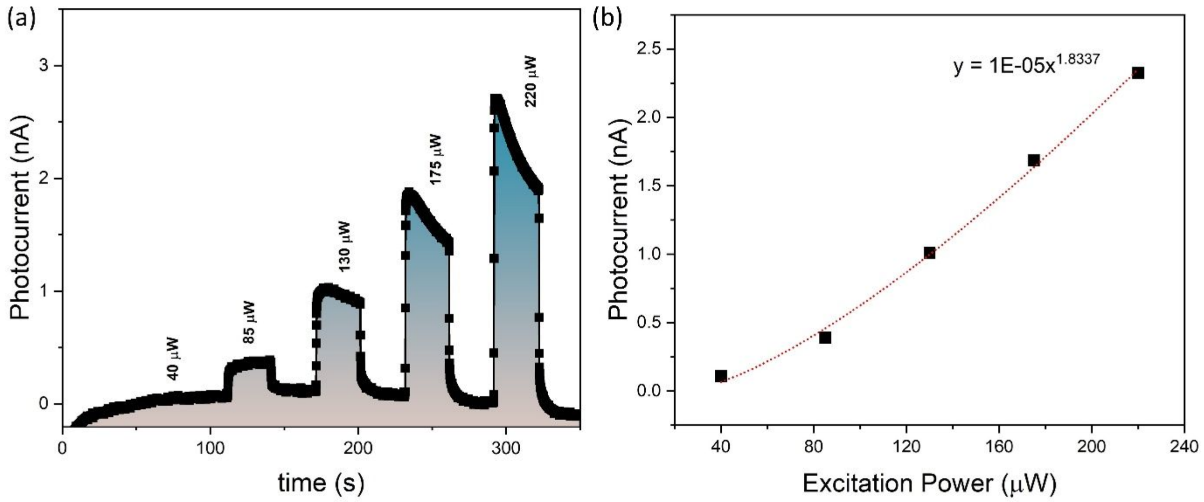

Figure S7. (a) HE photocurrents as function of illumination intensity and (b) fitting showing superlinear dependence.

Moreover, we have shown that SERS spectra (Figure S8) show no appreciable change in peak position and intensity upon heating, which confirms previous observations that the photocatalytic pATP conversion is not affected by thermophotonic processes.[7,8]

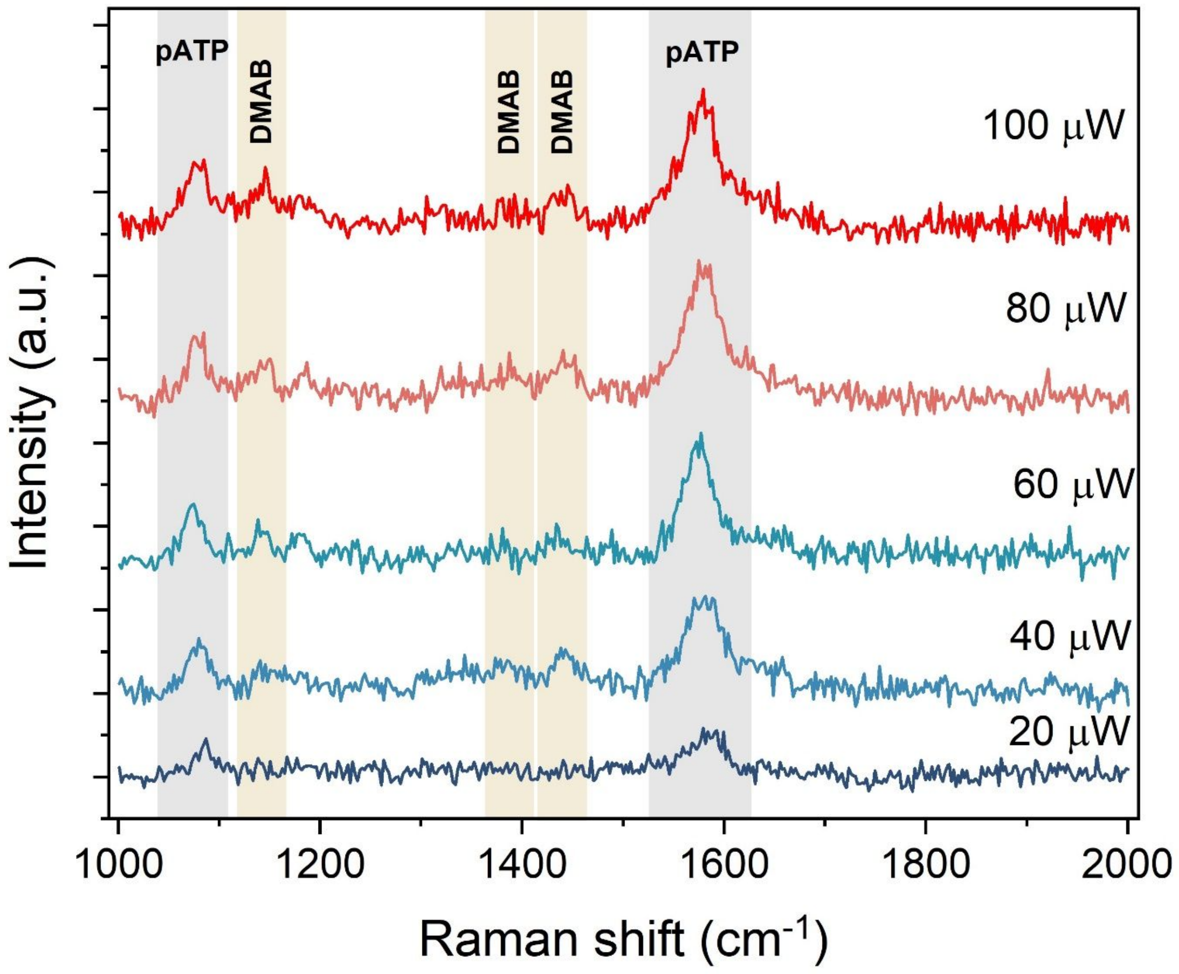

Figure S8. SERS spectra at gold rich side obtained at different excitation powers that show similar ratios of pATP and DMAB after 5-minute exposure.

7. Calculation of hot electron yield

The plasmon dissipation rate is composed of several contributions including the hot-electron loss.[9]

$$\gamma_{plasmon} = \gamma_{drude} + \gamma_{inter-band} + \gamma_{hot-electron}$$

The plasmonic dissipation rate was extracted from the SPR decay time τ_1 according

$$\gamma_{plasmon} = 1/\tau_1$$

The hot electron loss can then be used to calculate the generation rate of hot electrons according to

$$Rate_{hot-electron} = \frac{E_{plasmon}}{\hbar\omega}\frac{1}{\gamma_{plasmon} - (\gamma_{drude} + \gamma_{inter-band})}$$

where all other dissipation rates were treated as fitting parameters. The hot-electron generation rate is therefore reported in arbitrary units.

8. EIS and DOS spectra of gradient bimetallic alloy (Ag_xAu_{1-x})

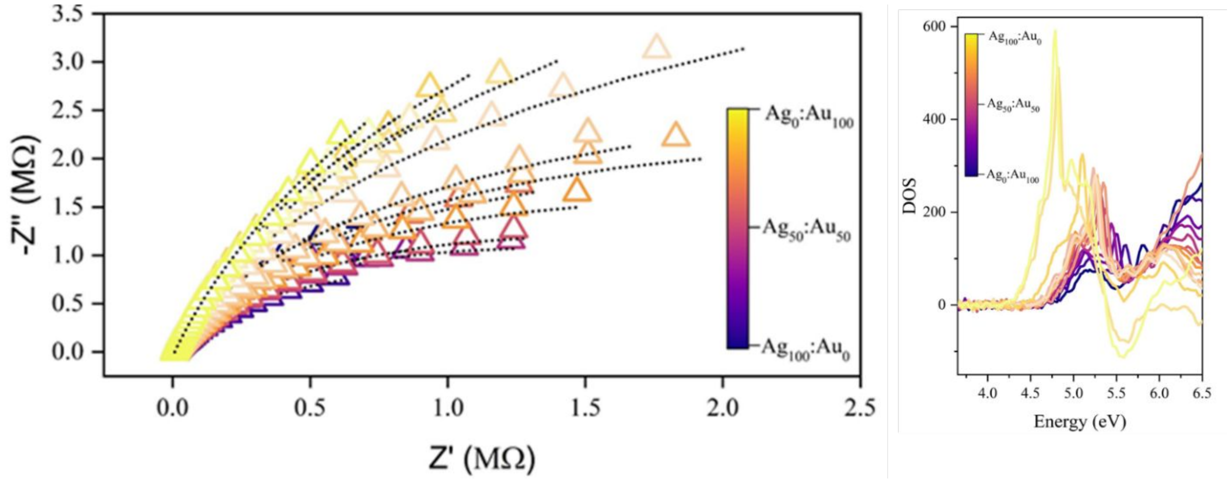

Figure S9. (a) Electrochemical impedance spectral (EIS) analysis. (b) Density of State (DOS) of bimetallic gradient alloy nanoparticles.

9. Photocatalytic CO_2 reduction reaction

We conduct CO_2RR by illuminating different locations of the alloyed sample (Figure S10(a)). Due to the large diameter of the light spot (~3mm) there is a substantial uncertainty in the precise composition contributing. However, a clear difference in CO yield (Figure S10(c)) can be seen when moving from the Au-rich side to the Ag-rich side that corroborates the difference in reactivity for HE catalysts.

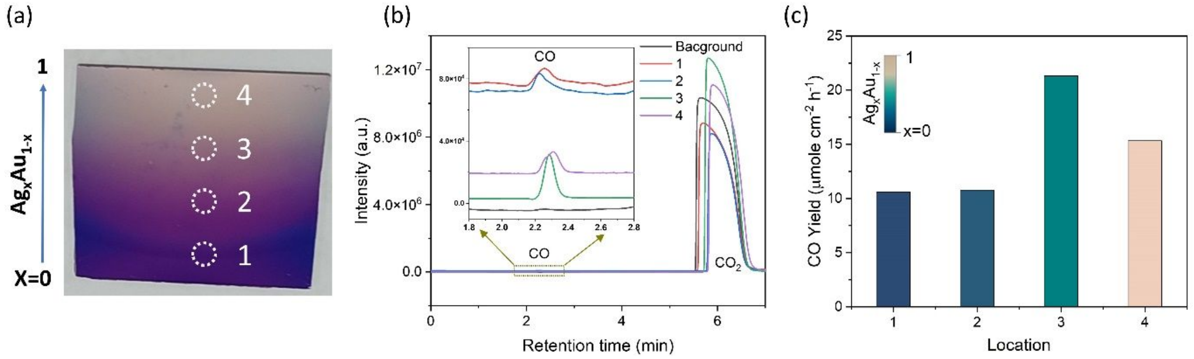

Figure S10. (a) Photograph of graded alloy sample used in photocatalytic CO2RR experiments, (b) gas chromatograph measurements of CO evolution at different locations, (c) extracted CO yield for different locations.

References

1. Yang, J.-L.; Wang, H.-J.; Zhang, H.; Tian, Z.-Q.; Li, J.-F. Probing hot electron behaviors by surface-enhanced raman spectroscopy. *Cell Reports Physical Science* **2020**, *1* (9).
2. Huang, Y.-F.; Zhu, H.-P.; Liu, G.-K.; Wu, D.-Y.; Ren, B.; Tian, Z.-Q. When the signal is not from the original molecule to be detected: chemical transformation of para-aminothiophenol on Ag during the SERS measurement. *Journal of the American Chemical Society* **2010**, *132* (27), 9244-9246.
3. Zhang, H.; Wei, J.; Zhang, X.-G.; Zhang, Y.-J.; Radjenovica, P. M.; Wu, D.-Y.; Pan, F.; Tian, Z.-Q.; Li, J.-F. Plasmon-induced interfacial hot-electron transfer directly probed by Raman spectroscopy. *Chem* **2020**, *6* (3), 689-702.
4. Verma, R.; Sharma, G.; Polshettiwar, V. The paradox of thermal vs. non-thermal effects in plasmonic photocatalysis. Nature Communications **2024**, 15 (1), 7974.
5. Karnetzky, C.; Zimmermann, P.; Trummer, C.; Duque Sierra, C.; Wörle, M.; Kienberger, R.; Holleitner, A. Towards femtosecond on-chip electronics based on plasmonic hot electron nano-emitters. Nature communications 2018, 9 (1), 2471.
6. Han, Q.; Zhang, C.; Gao, W.; Han, Z.; Liu, T.; Li, C.; Wang, Z.; He, E.; Zheng, H. Ag-Au alloy nanoparticles: Synthesis and in situ monitoring SERS of plasmonic catalysis. Sensors and Actuators B: Chemical 2016, 231, 609-614.
7. Alessandri, I. 4-Aminothiophenol Photodimerization Without Plasmons. Angewandte Chemie International Edition 2022, 61 (28), e202205013.
8. Guo, J.; Zhang, Y.; Shi, L.; Zhu, Y.; Mideksa, M. F.; Hou, K.; Zhao, W.; Wang, D.; Zhao, M.; Zhang, X. Boosting hot electrons in hetero-superstructures for plasmon-enhanced catalysis. Journal of the American Chemical Society 2017, 139 (49), 17964-17972.
9. Besteiro, L. V., Kong, X. T., Wang, Z., Hartland, G., & Govorov, A. O. Understanding hot-electron generation and plasmon relaxation in metal nanocrystals: Quantum and classical mechanisms. Acs Photonics **2017**, 4(11), 2759-2781.
